# Supplementary material for: Prognostic significance of BIRC7/Livin, Bcl-2, p53, Annexin V, PD-L1, DARC, MSH2 and PMS2 in colorectal cancer treated with FOLFOX chemotherapy with or without aspirin
Source: PLoS One. 2021 Jan 19;16(1):e0245581. doi: 10.1371/journal.pone.0245581 (PMC7815153; doi:10.1371/journal.pone.0245581)
Supplement: S1 Appendix — (DOCX) [file pone.0245581.s001.docx]

S1 Appendix: Tabular Presentation of Treatment Composition in the Rat-bearing CRC

| Groups | Number of Albino Rats | Tumor induction, Treatment agents and methods | administration Route |
| --- | --- | --- | --- |
| Group 1 | 10 | Water and saline during cancer induction and treatment in other Rats respectively | Intra rectal |
| Group 2 | 10 | 2 mg/kg NMU alone (60 days) | Intra rectal |
| Group 3 | 10 | 2 mg/kg NMU (60 days) + 25 mg/kg Aspirin | Intra rectal and oral respectively |
| Group 4 | 10 | 2 mg/kg NMU (60 days) + 25mg/kg Aspirin (2 days) + 7mg/kg Folinic acid + 3 mg/kg Oxaliplatin + 50 mg/kg 5-FU (3 days) | Intra rectal, oral, and intravenous respectively |
| Group 5 | 10 | 2 mg/kg NMU (60 days) + 25mg/kg Aspirin + 7mg/kg Folinic acid + 3 mg/kg Oxaliplatin + 50 mg/kg 5-FU (5 days) | Intra rectal, oral, and intravenous respectively |
| Group 6 | 10 | 2 mg/kg NMU (60 days) + 7mg/kg folinic acid alone (5 days) | Intra rectal, oral, and intravenous |
| GROUP 7 | 10 | 2 mg/kg NMU (40 days) + 7mg/kg folinic acid alone (5 days) | Intra rectal, oral, and intravenous |
| GROUP 8 | 10 | 2 mg/kg NMU (40 days) + 3 mg/kg oxaliplatin alone (5 days) | Intra rectal, oral, and intravenous |
