# Supplementary material for: Prognostic significance of BIRC7/Livin, Bcl-2, p53, Annexin V, PD-L1, DARC, MSH2 and PMS2 in colorectal cancer treated with FOLFOX chemotherapy with or without aspirin
Source: PLoS One. 2021 Jan 19;16(1):e0245581. doi: 10.1371/journal.pone.0245581 (PMC7815153; doi:10.1371/journal.pone.0245581)

ORIGINAL BLOTS

NB: Our interest for this article was Aspirin alone, thus we did not include the Aspirin analogs used in our overall study in this article, rather only Aspirin blots. However, for clarity, we have indicated the entire blots of aspirin and other Analogues in this study

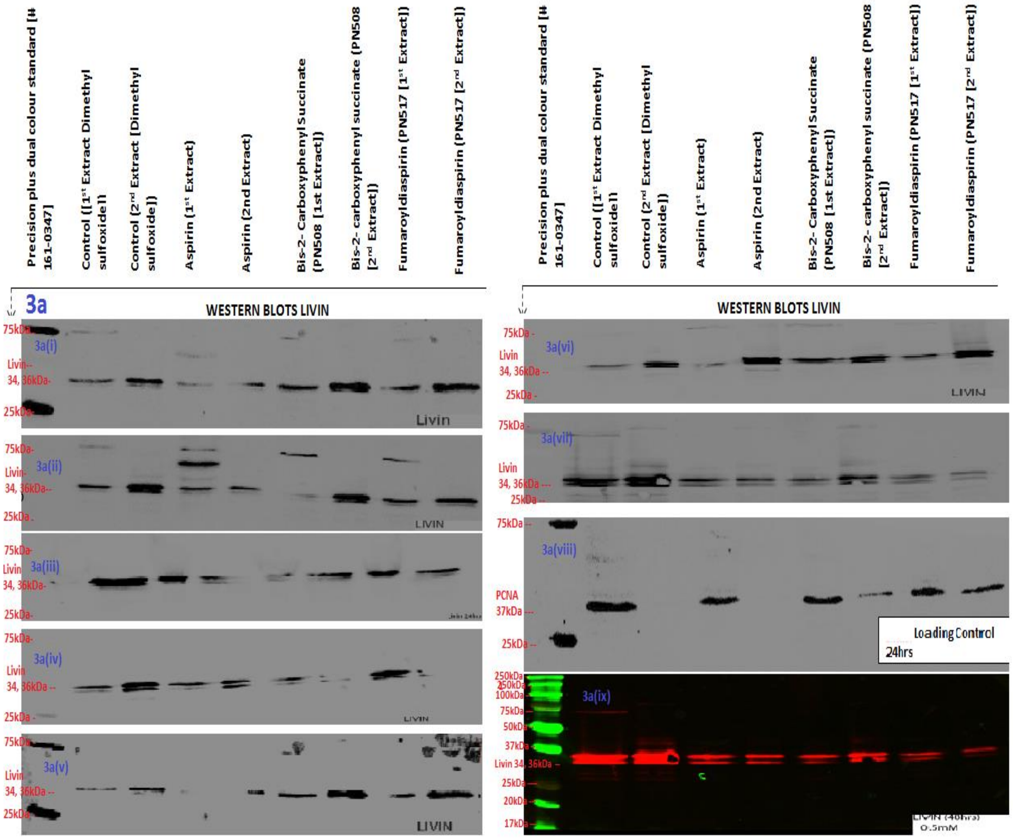

Figure a: Immunoblot analysis demonstrating considerable reduced expression of baculoviral IAP repeat-containing protein 7 (Livin) in colorectal cancer by Acetylsalicylic acid (Aspirin), Bis-2 carboxyphenyl succinate (PN508) and Fumaroyldiaspirin (PN517); figure 3a(I)-3a(VII) show reduced expression of livin protein (with molecular weight 34, 36kDa) in colorectal cancer cells (SW480 cell line) on treatment with 0.5mM drugs (Aspirin, PN508 & PN517 respectively) for 24hrs as compared to the control (DMS0) treated cells. The positive action of the drugs (Aspirin, PN508 & PN517) on the colorectal cancer cell is a new breakthrough towards combating these dreaded cancer (CRC) and possibly many other cancer that have livin protein implication in their pathogenesis. **Figure a1 (VIII)** is PCNA loading control to indicate equal loading of the protein. **Figure a2** is a coloured image of the 3a (xii) indicating clearly the positions of bands by the precision plus dual colour protein standard.

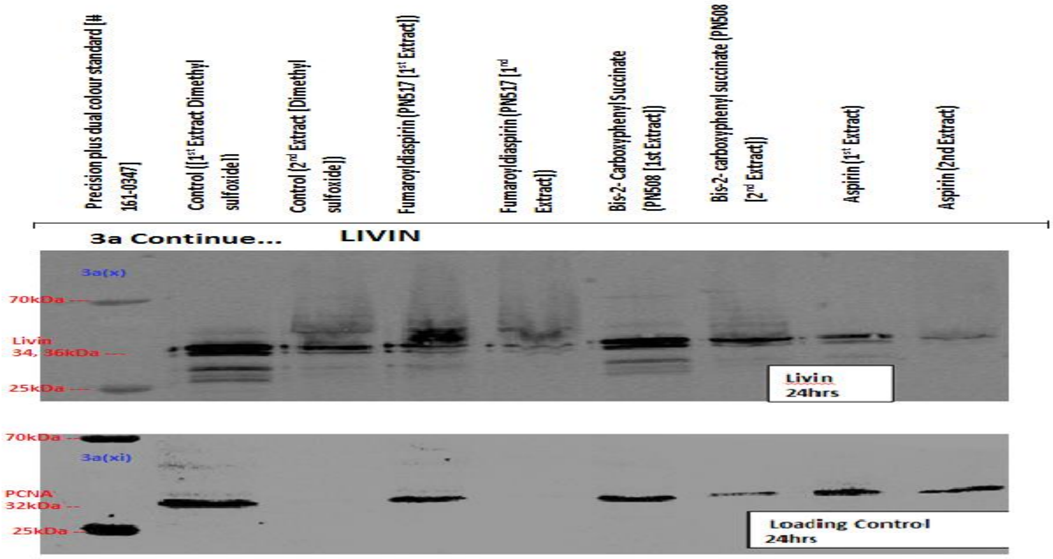

Figure b: Immunoblot analysis demonstrating considerable reduced expression in livin protein in colorectal cancer cells after treatment with 0.5mM drugs (PN517, PN508 & Aspirin, respectively) for 24hrs as compared to the control (DMS0).

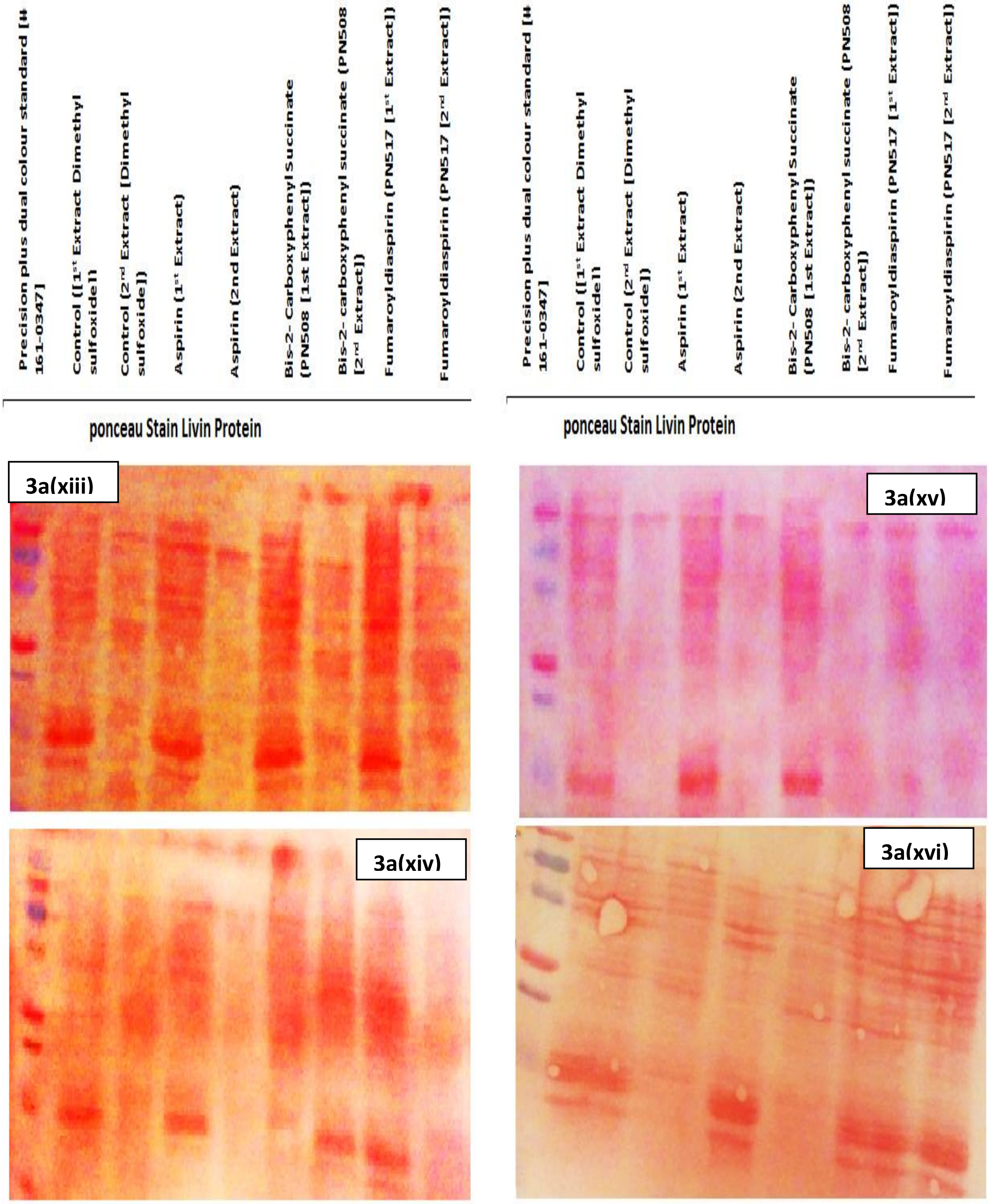

Figure 3c (XIII, XIV, XV and XVI) show ponceau stain membranes of protein extracts (for livin protein) from the colorectal cancer treated with Acetylsalicylic acid (Aspirin), Bis-2 carboxyphenyl succinate (PN508) and Fumaroyldiaspirin (PN517)

FINAL BLOT USED FOR PUBLICATION IS AS BELOW

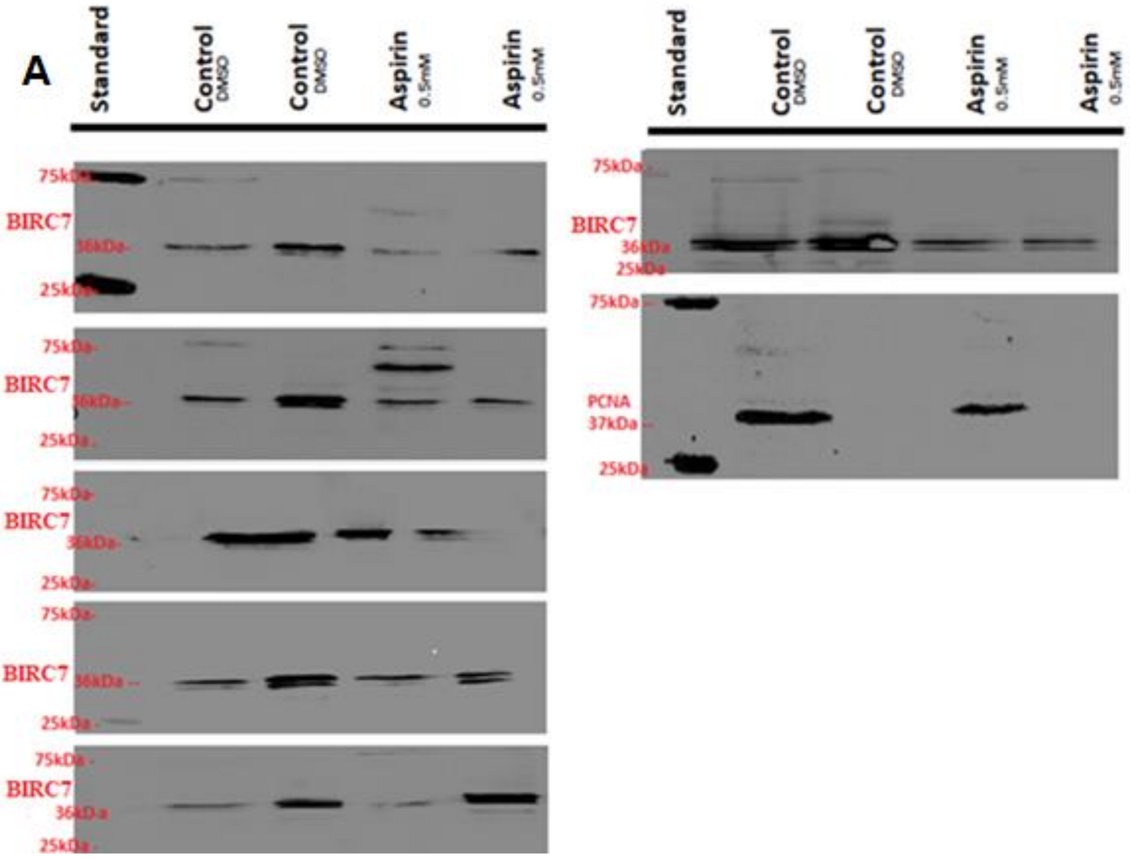

Supplement: S1 Raw images — (PDF) [file pone.0245581.s002.pdf]
