# Supplementary material for: Prognostic significance of BIRC7/Livin, Bcl-2, p53, Annexin V, PD-L1, DARC, MSH2 and PMS2 in colorectal cancer treated with FOLFOX chemotherapy with or without aspirin
Source: PLoS One. 2021 Jan 19;16(1):e0245581. doi: 10.1371/journal.pone.0245581 (PMC7815153; doi:10.1371/journal.pone.0245581)
Supplement: Attachment — Submitted filename: SIGNED REBUTTAL LETTER.pdf [file pone.0245581.s003.pdf]

DEPARTMENT OF PATHOLOGY

(MORBID ANATOMY)

AHMADU BELLO UNIVERSITY

ZARIA - NIGERIA

Vice Chancellor: Prof. Ibrahim Garba, B.Sc, (Hons) Geology, M.Sc (Mineral Exploration) Ph. D Geology (London), D.I.C., FNMGS

Head of Department: Dr. Almustapha A. Liman *MBBS (ABU), FMCPath, FICS, FCPath (ECSA)*

Date: 31st October, 2020

The Guest Editors

Drs. Joe Ramos, Shengyu Yang, Helen Fillmore, and Tobias Zech

PLOS ONE Cancer Metastasis

Dear Guest Editors,

Rebuttal letter to the Editor that responds to each point raised by the academic editor and reviewers:

Addressing points raised by the Editor:

1- The article is here edited to PLOS ONE's style requirements.

2- Additional information about the patient records used in in the study, including as regard date range (month and year) during which patients' medical records were accessed and b) the date range (month and year) during which patients whose medical records were selected for this study sought treatment were addressed as follows:

The series included 23 mucinous CRC and 69 CRC NOS FFPE tissue blocks selected from January 2009 to December 2017. Among these cases, 16 patients received neoadjuvant FOLFOX chemotherapy from September 2010 to December 2013 after a biopsy-confirmed histological diagnosis of CRC

3- Regarding animal care as per editorial guidelines, the following statements were added in the appropriate place and clarified:

To minimize the rats suffering and distress, we worked as a team with veterinarians and animal care personnel from the ethic committee and staff members who are experienced in handling laboratory animals at all stage of the experiment and throughout the study period for appropriate monitoring and guidance.

The rats were purchased from Animal facility of the Faculty of Pharmaceutical Sciences, Ahmadu Bello University Zaria Nigeria.

The ethics committee at ABU (ABU committee on Animal use and care-https://abu.edu.ng/animal-use/) who reviewed and approved this study contains animal welfare experts from Faculty of Veterinary Medicine of the University.

The collection of the blood was done by animal care expert under the supervision of senior veterinarian while restraining the rat manually and minimising the time of restrain and amount of blood collected so as to reduce stress and pain to the rats.

4- The word “Caucasian” was updated to “Whites” in the publication.

5- In the Methods section, the source, product number and lot numbers of the N-Methyl-N-Nitrosourea (NMU) used in the animal experiments in our study uodated as follows:

N-Methyl-N-Nitrosourea (NMU; Shijiazhuang Aopharm Medical Technology Co., Ltd, China #684-93-5)

6- The source, product number/ lot numbers of the primary antibodies used in the histology analysis for our study were all provided as appropriate in the manuscript, see below:

Anti-BIRC7 polyclonal antibodies from Antibodies-online (Aachen, Germany; ABIN358607; 1:80 dilution and ABIN672561; 1:100), Anti-Annexin V polyclonal antibody from Antibodies-online (Aachen Germany; ABIN4964891; 1:80 dilution), Anti-PD-L1 (CD274) monoclonal antibody from Antibodies-online (Aachen, Germany; ABIN5027498; 5 µg/mL), Anti-DARC polyclonal antibody from Antibodies-online (Aachen Germany; ABIN2821184; 1:50), Anti-MSH2 polyclonal antibody from Antibodies-online (Aachen Germany; ABIN3185692; 1:100), Anti-PMS2 polyclonal antibody from Antibodies-online (Aachen Germany; ABIN5546942; 1;30), Anti-Bcl-2 monoclonal antibody from Genemed Biotechnologies, Inc. (CA, USA; Clone Bcl-2-100; 1:60 dilution) and Anti-p53 monoclonal antibody from Genemed Biotechnologies, Inc. (CA, USA; Clone BP-53-12; 1:60 dilution).

Anti-BIRC7 antibody from Antibodies-online (Aachen, Germany, ABIN672561; 1:100 dilution)

7- Additional information regarding statistical analysis were updated in the method section

8- Scale bars were update in the microscopy and reflected in the figure legend.

9- Tables were included as part of the manuscript

Addressing points raised by Reviewer 1:

The 92 FFPE CRC patients samples used have only 16 patients treated with neoadjuvant chemotherapy as indicated in the method. These patients were not treated Aspirin. Only animal study was subjected to Aspirin. However, we envisage that there will be room to go ahead with human trial with Aspirin plus FOLFOX in future in African patients. Limited funding for translational research in Africa and the smaller sample size with limited number of patients who had received chemotherapy makes it difficult to analyse the relation between MSI, tumour stage and other biomarkers. However, we look forward to expanding the sample size to give room for these analyses when funding becomes available. Other parameters such as age location were updated in the manuscript.

Addressing points raised by Reviewer 2:

The sentence in the third line of the abstract was re-written as follows:

These include overexpression of pro-apoptotic and anti-apoptotic proteins (including p53 and PD-L1; BIRC7/Livin and Bcl-2), chemokine receptors (including DARC), and dysregulation of DNA mismatch repair proteins (including MSH2 and PMS2).

The points raised in introduction were revised as follows:

1- Thus, caspase-3 -7 and -9 and the second mitochondria-derived activator of caspases (SMAC/DIABLO) are crucial interacting partners of BIRC7/Livin (16)

2- The p53 protein is important for initiation of the apoptotic stimuli during anticancer therapy by sensing DNA damage and activating a series of cellular processes.

3- Annexin V, an important marker for detection apoptotic cells by its ability to bind to phosphatidylserine (outer leaflet of the plasma membrane), has been reported to stimulate immunogenicity of tumor cells (25-26).

4- Aspirin inhibits cyclooxygenase (COX), reducing PGE2 production and inducing a pro-tumour inflammatory profile -Aspirin may revert this towards an important anti-cancer immune pathway and possibly serve as an adjuvant for immune checkpoint therapy [36-37].

All points raised by the reviewer in the result section were addressed accordingly. The numbers (N) were indicated in the figures appropriately.

Table 2, 3 and 4 were all placed appropriately for easy identification.

The small sample size for the human CRC treated for neoadjuvant FOLFOX limit our capacity to explore the reason behind expression pattern of the proteins in human subjects. However, we will make to increase sample size in future studies.
We are of the view that treatment with Folinic acid alone without the addition of Oxaliplatin and 5-FU may have increase toxicity to cells generally.

All other points raised were addressed.

In the discussion, the sentence was re-written as follows:
Cancer chemotherapeutics such as FOLFOX may induce direct damage to cancer cells with increase apoptosis and equally reduce drug sensitivity which may lead to treatment failure.

I am attaching herewith our manuscript for you kind consideration.

Yours faithfully,

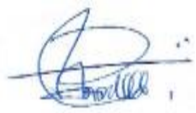

Faruk Mohammed
Study Principal Investigator
Phone: +234 803 220 0648
Email: fmohammed@abu.edu.ng elfarukpath11@yahoo.com
