# Supplementary material for: Prognostic significance of BIRC7/Livin, Bcl-2, p53, Annexin V, PD-L1, DARC, MSH2 and PMS2 in colorectal cancer treated with FOLFOX chemotherapy with or without aspirin
Source: PLoS One. 2021 Jan 19;16(1):e0245581. doi: 10.1371/journal.pone.0245581 (PMC7815153; doi:10.1371/journal.pone.0245581)
Supplement: Attachment — Submitted filename: SIGNED 2nd REBUTTAL LETTER FARUK PLOS ONE 18.12.2020.pdf [file pone.0245581.s004.pdf]

// DEPARTMENT OF PATHOLOGY
(MORBID ANATOMY)
AHMADU BELLO UNIVERSITY
ZARIA - NIGERIA
Vice Chancellor: Prof. Ibrahim Garba, B.Sc, (Hons) Geology, M.Sc (Mineral Exploration) Ph. D Geology (London), D.I.C., FNMGS
Head of Department: Dr. Almustapha A. Liman *MBBS (ABU), FMCPath, FICS, FCPath (ECSA)*

Date: 18st December, 2020

The Guest Editors

Drs. Joe Ramos, Shengyu Yang, Helen Fillmore, and Tobias Zech

PLOS ONE Cancer Metastasis

Dear Guest Editors,

Second Rebuttal letter to the Editor that responds to each point raised by the academic editor and reviewers:

Addressing points raised by Reviewer #2:

1-The sentence in the third line of the abstract was re-written for second time as follows based on reviewers guide and suggestion to separate each class of gene(s) by bracket:

Various cellular processes are associated with evasion of apoptosis. These include overexpression of pro-apoptotic protiens (including p53 and PD-L1), anti-apoptotic proteins (BIRC7/Livin and Bcl-2), chemokine receptors (including DARC), and dysregulation of DNA mismatch repair proteins (including MSH2 and PMS2).

2- In the discussion, the sentence was re-written for second time to address reviewer's point as follows (This clarify our what we want to say):

Cancer chemotherapy regimens such as FOLFOX have been shown to significantly increased treatment efficacy and improved survival especially in metastatic CRC however, this treatment modality may be accompanied by complications such as myelotoxicity, neurotoxicity, non-alcoholic fatty liver disease and sinusoid obstruction syndrome (46).

3- The rational for testing aspirin in the first place is clearly indicated as per reviewer's suggestions and for clarity as follows:

Aspirin has been shown to be effective in preventing recurrence, decreasing risk of metastasis post curative therapy and primary prevention of CRC (47). However, complications from Aspirin use such as occult gastrointestinal bleeding, epistaxis extra and intra-cranial bleeding which are very rare, may resolve without much ramification (48). There is limited research that explores the mechanism through which aspirin alone and in combination with other chemotherapeutics exert anti-cancer effect especially in Black Africans with CRC. Thus, this strengthen the rational for testing Aspirin plus FOLFOX towards identifying biomarkers and clinical characteristics which may predict the benefit of Aspirin use for targeted invention in CRC management.

4-The figures are well re-arranged as suggested.

5- Dot plots were presented as advised.

I am attaching herewith our manuscript for you kind consideration.

Yours faithfully,

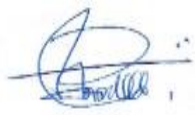

Faruk Mohammed

Study Principal Investigator

Phone: +234 803 220 0648

Email: fmohammed@abu.edu.ng elfarukpath11@yahoo.com
